# Supplementary material for: The Influence of Daily Temperature Fluctuation on the Efficacy of Bioinsecticides on Spotted Wing Drosophila Larvae
Source: Insects. 2022 Dec 31;14(1):43. doi: 10.3390/insects14010043 (PMC9866168; doi:10.3390/insects14010043)
Supplement: Supplementary file 1 [file insects-14-00043-s001.zip › insects-2064955-supplementary.pdf]

The influence of daily temperature fluctuation on the efficacy of bioinsecticides on Spotted Wing Drosophila larvae

Maristella Mastore[1], Silvia Quadroni[2], Alberto Rezzonico[1], Maurizio Francesco Brivio[1*]

[1]Lab. of Environmental Entomology and Parasitology, Dept. of Theoretical and Applied Sciences, University of Insubria, 21100 Varese, Italy
[2]Lab. of Ecology, Dept. of Theoretical and Applied Sciences, University of Insubria, 21100 Varese, Italy

*Corresponding Author: Maurizio Francesco Brivio – e-mail: maurizio.brivio@uninsubria.it

SUPPLEMENTARY MATERIAL

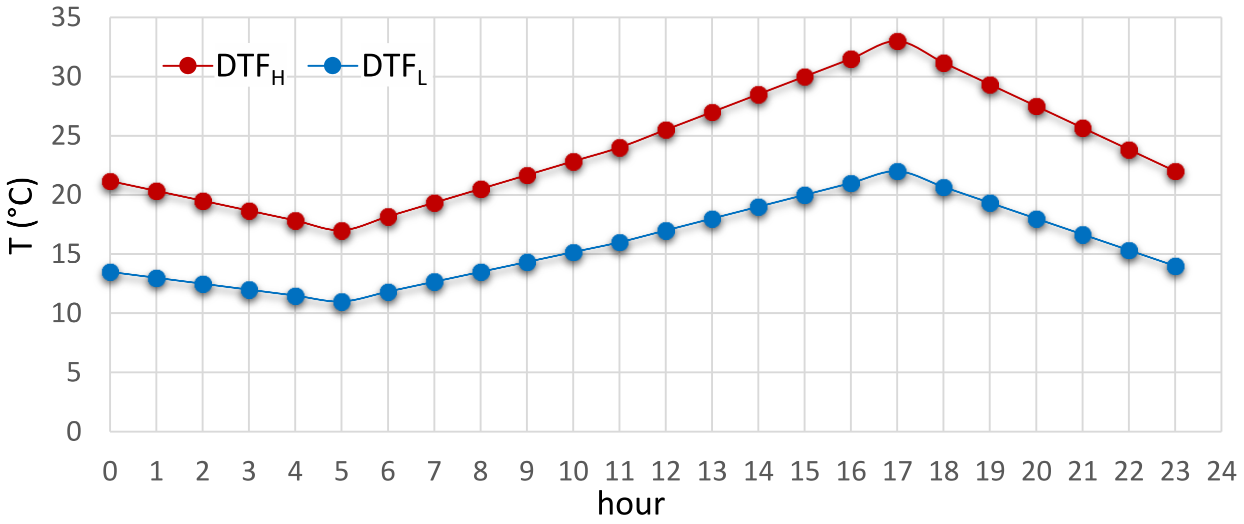

Figure S1. Thermal variation set in the climate chamber over 24 hours in the two temperature ranges, DTF$_L$ 11-22 °C and DTF$_H$ 17-33 °C.

Table S1. Pairwise comparisons of SWD mortality (estimated marginal means) after logistic regression (logit model), between different concentrations at the same time and temperature condition. Significant p-values are shown in bold. Cnt = control, C_1-C_5 concentrations of Btk: 640, 1900, 3800, 7600 and 15200 IU/mL, nematodes: 100, 200, 400, 800 and 1600 IJ/mL

		**Btk**				**Sc**				**Sf**				**Hb**			
		**24 h**		**48 h**		**24 h**		**48 h**		**24 h**		**48 h**		**24 h**		**48 h**	
		**Z**	**p**	**Z**	**p**	**Z**	**p**	**Z**	**p**	**Z**	**p**	**Z**	**P**	**Z**	**p**	**Z**	**p**
**CT**	**Cnt vs C_1**	0.7	1.000	2.4	0.872	4.0	**0.031**	3.7	0.103	3.8	**0.041**	6.7	**< 0.0001**	0.0	1.000	0.0	1.000
	**Cnt vs C_2**	5.5	**< 0.0001**	5.3	**0.0002**	5.7	**< 0.0001**	5.7	**< 0.0001**	6.2	**< 0.0001**	7.5	**< 0.0001**	0.9	1.000	0.3	1.000
	**Cnt vs C_3**	7.8	**< 0.0001**	9.8	**< 0.0001**	8.2	**< 0.0001**	10.9	**< 0.0001**	7.1	**< 0.0001**	8.1	**< 0.0001**	1.8	0.982	1.0	1.000
	**Cnt vs C_4**	10.6	**< 0.0001**	12.5	**< 0.0001**	12.6	**< 0.0001**	14.8	**< 0.0001**	8.0	**< 0.0001**	8.8	**< 0.0001**	3.2	0.191	2.4	0.721
	**Cnt vs C_5**	15.1	**< 0.0001**	20.8	**< 0.0001**	23.2	**< 0.0001**	23.3	**< 0.0001**	8.5	**< 0.0001**	9.8	**< 0.0001**	5.9	**< 0.0001**	4.0	**0.020**
	**C_1 vs C_2**	4.8	**0.002**	2.8	0.636	1.6	1.000	2.1	0.983	2.4	0.710	0.8	1.000	0.9	1.000	0.3	1.000
	**C_1 vs C_3**	7.2	**< 0.0001**	7.3	**< 0.0001**	4.2	**0.021**	7.3	**< 0.0001**	3.4	0.141	1.4	1.000	1.8	0.982	1.0	1.000
	**C_1 vs C_4**	10.0	**< 0.0001**	10.0	**< 0.0001**	8.6	**< 0.0001**	11.1	**< 0.0001**	4.3	**0.009**	2.1	0.919	3.2	0.191	2.4	0.721
	**C_1 vs C_5**	14.4	**< 0.0001**	18.3	**< 0.0001**	19.2	**< 0.0001**	19.6	**< 0.0001**	4.7	**0.001**	3.1	0.233	5.9	**< 0.0001**	4.0	**0.020**
	**C_2 vs C_3**	2.4	0.900	4.5	**0.006**	2.5	0.826	5.2	**0.0003**	0.9	1.000	0.5	1.000	0.9	1.000	0.7	1.000
	**C_2 vs C_4**	5.2	**0.0003**	7.2	**< 0.0001**	7.0	**< 0.0001**	9.0	**< 0.0001**	1.8	0.980	1.3	1.000	2.3	0.802	2.2	0.875
	**C_2 vs C_5**	9.7	**< 0.0001**	15.5	**< 0.0001**	17.6	**< 0.0001**	17.5	**< 0.0001**	2.3	0.812	2.3	0.786	5.0	**0.001**	3.8	**0.046**
	**C_3 vs C_4**	2.8	0.649	2.7	0.714	4.4	**0.007**	3.8	0.065	0.9	1.000	0.7	1.000	1.4	0.999	1.4	0.999
	**C_3 vs C_5**	7.3	**< 0.0001**	11.0	**< 0.0001**	15.0	**< 0.0001**	12.3	**< 0.0001**	1.4	0.999	1.8	0.982	4.1	**0.015**	3.0	0.301
	**C_4 vs C_5**	4.5	**0.006**	8.3	**< 0.0001**	10.6	**< 0.0001**	8.5	**< 0.0001**	0.5	1.000	1.1	1.000	2.7	0.550	1.6	0.996

		Btk				Sc				Sf			
		**24 h**		**48 h**		**24 h**		**48 h**		**24 h**		**48 h**	
		**Z**	**p**	**Z**	**p**	**Z**	**p**	**Z**	**p**	**Z**	**p**	**Z**	**p**
	**Cnt vs C_1**	3.5	0.150	4.6	**0.003**	3.2	0.324	4.8	**0.002**	1.4	0.999	3.6	0.077
	**Cnt vs C_2**	5.4	**< 0.0001**	6.5	**< 0.0001**	3.9	**0.044**	5.4	**< 0.0001**	4.4	**0.006**	5.2	**0.0003**
	**Cnt vs C_3**	6.9	**< 0.0001**	10.2	**< 0.0001**	5.0	**0.001**	6.2	**< 0.0001**	5.0	**0.0005**	7.0	**< 0.0001**
	**Cnt vs C_4**	8.6	**< 0.0001**	11.9	**< 0.0001**	5.8	**< 0.0001**	7.8	**< 0.0001**	5.9	**< 0.0001**	7.7	**< 0.0001**
	**Cnt vs C_5**	13.5	**< 0.0001**	17.5	**< 0.0001**	6.4	**< 0.0001**	10.0	**< 0.0001**	7.0	**< 0.0001**	8.4	**< 0.0001**
	**C_1 vs C_2**	1.9	0.995	1.8	0.998	0.7	1.000	0.6	1.000	3.0	0.322	1.6	0.996
	**C_1 vs C_3**	3.4	0.234	5.5	**< 0.0001**	1.8	0.998	1.4	1.000	3.6	0.067	3.5	0.103
**DTF_L**	**C_1 vs C_4**	5.0	**0.001**	7.3	**< 0.0001**	2.6	0.774	3.0	0.463	4.5	**0.003**	4.2	**0.012**
	**C_1 vs C_5**	10.0	**< 0.0001**	12.9	**< 0.0001**	3.2	0.336	5.2	**0.0003**	5.6	**< 0.0001**	4.8	**0.001**
	**C_2 vs C_3**	1.5	1.000	3.7	0.094	1.0	1.000	0.8	1.000	0.6	1.000	1.9	0.968
	**C_2 vs C_4**	3.1	0.366	5.5	**< 0.0001**	1.9	0.996	2.4	0.876	1.5	0.997	2.6	0.619
	**C_2 vs C_5**	8.1	**< 0.0001**	11.1	**< 0.0001**	2.5	0.870	4.6	**0.004**	2.6	0.590	3.2	0.188
	**C_3 vs C_4**	1.7	0.999	1.8	0.999	0.8	1.000	1.6	1.000	0.9	1.000	0.7	1.000
	**C_3 vs C_5**	6.7	**< 0.0001**	7.4	**< 0.0001**	1.4	1.000	3.8	0.068	2.0	0.948	1.4	1.000
	**C_4 vs C_5**	5.0	**0.001**	5.6	**< 0.0001**	0.6	1.000	2.2	0.969	1.1	1.000	0.7	1.000

		Btk				Sc				Hb			
		24 h		48 h		24 h		48 h		24 h		48 h	
		Z	p	Z	p	Z	p	Z	p	Z	p	Z	p
DTF_H	Cnt vs C_1	4.9	**0.001**	5.0	**0.001**	1.8	0.997	7.6	**< 0.0001**	0.2	1.000	0.3	1.000
	Cnt vs C_2	6.6	**< 0.0001**	7.1	**< 0.0001**	5.4	**0.0001**	8.3	**< 0.0001**	0.6	1.000	0.9	1.000
	Cnt vs C_3	9.5	**< 0.0001**	11.2	**< 0.0001**	6.1	**< 0.0001**	9.0	**< 0.0001**	1.4	0.999	2.1	0.896
	Cnt vs C_4	10.5	**< 0.0001**	13.1	**< 0.0001**	7.3	**< 0.0001**	14.0	**< 0.0001**	3.0	0.335	3.0	0.300
	Cnt vs C_5	15.1	**< 0.0001**	17.9	**< 0.0001**	12.4	**< 0.0001**	16.4	**< 0.0001**	3.9	**0.030**	4.2	**0.012**
	C_1 vs C_2	1.7	0.999	2.1	0.974	3.6	0.140	0.7	1.000	0.3	1.000	0.5	1.000
	C_1 vs C_3	4.7	**0.003**	6.1	**< 0.0001**	4.3	**0.013**	1.4	1.000	1.2	1.000	1.8	0.983
	C_1 vs C_4	5.6	**< 0.0001**	8.1	**< 0.0001**	5.5	**< 0.0001**	6.4	**< 0.0001**	2.7	0.489	2.7	0.545
	C_1 vs C_5	10.2	**< 0.0001**	12.8	**< 0.0001**	10.6	**< 0.0001**	8.8	**< 0.0001**	3.7	0.059	3.8	**0.039**
	C_2 vs C_3	3.0	0.515	4.0	**0.035**	0.7	1.000	0.7	1.000	0.9	1.000	1.3	1.000
	C_2 vs C_4	3.9	0.051	5.9	**< 0.0001**	1.9	0.994	5.7	**< 0.0001**	2.4	0.747	2.1	0.886
	C_2 vs C_5	8.5	**< 0.0001**	10.7	**< 0.0001**	7.0	**< 0.0001**	8.1	**< 0.0001**	3.3	0.153	3.3	0.167
	C_3 vs C_4	0.9	1.000	1.9	0.994	1.2	1.000	5.0	**0.001**	1.5	0.998	0.9	1.000
	C_3 vs C_5	5.6	**< 0.0001**	6.7	**< 0.0001**	6.3	**< 0.0001**	7.4	**< 0.0001**	2.4	0.712	2.0	0.931
	C_4 vs C_5	4.6	**0.004**	4.8	**0.002**	5.1	**0.001**	2.3	0.921	0.9	1.000	1.1	1.000

Table S2. Pairwise comparisons of SWD mortality (estimated marginal means) after logistic regression (logit model), between different times at the same concentration and temperature condition. Significant p-values are shown in bold. Cnt = control, C_1-C_5 concentrations of Btk: 640, 1900, 3800, 7600 and 15200 IU/mL, nematodes: 100, 200, 400, 800 and 1600 IJ/mL

		**Btk**		**Sc**		**Sf**		**Hb**	
		**24 h vs 48 h**		**24 h vs 48 h**		**24 h vs 48 h**		**24 h vs 48 h**	
		**Z**	**p**	**Z**	**p**	**Z**	**p**	**Z**	**p**
**CT**	**Cnt**	2.6	0.769	2.9	0.564	4.5	**0.004**	3.7	0.059
	**C_1**	4.4	**0.008**	2.5	0.840	7.4	**< 0.0001**	3.7	0.059
	**C_2**	2.4	0.887	3.0	0.508	5.7	**< 0.0001**	3.0	0.305
	**C_3**	4.5	**0.005**	5.6	**< 0.0001**	5.4	**0.0001**	2.9	0.393
	**C_4**	4.4	**0.007**	5.0	**0.001**	5.2	**0.0002**	2.9	0.394
	**C_5**	8.3	**< 0.0001**	2.9	0.516	5.8	**< 0.0001**	1.8	0.981
**DTF_L**	**Cnt**	0.3	1.000	0.4	1.000	0.4	1.000		
	**C_1**	1.4	1.000	2.0	0.986	2.6	0.588		
	**C_2**	1.4	1.000	1.9	0.996	1.2	1.000		
	**C_3**	3.6	0.122	1.6	1.000	2.5	0.701		
	**C_4**	3.7	0.103	2.4	0.876	2.3	0.834		
	**C_5**	4.3	**0.012**	4.0	**0.033**	1.8	0.974		
**DTF_H**	**Cnt**	1.5	1.000	1.8	0.997			1.5	0.998
	**C_1**	1.6	1.000	7.6	**< 0.0001**			1.7	0.993
	**C_2**	2.0	0.989	4.8	**0.002**			1.8	0.976
	**C_3**	3.1	0.421	4.7	**0.002**			2.2	0.855
	**C_4**	4.1	**0.031**	8.6	**< 0.0001**			1.6	0.996
	**C_5**	4.2	**0.017**	5.8	**< 0.0001**			1.8	0.981

Table S3. Pairwise comparisons of SWD mortality (estimated marginal means) after logistic regression (logit model), between different temperature condition at the same time and concentration. Significant p-values are shown in bold. Cnt = control, C_1-C_5 concentrations of Btk: 640, 1900, 3800, 7600 and 15200 IU/mL, nematodes: 100, 200, 400, 800 and 1600 IJ/mL

			Btk		Sc		Sf		Hb	
			Z	p	Z	p	Z	p	Z	p
CT vs DTF_L	24 h	**Cnt**	0.7	1.000	0.4	1.000	1.5	0.997		
		**C_1**	3.5	0.150	0.4	1.000	0.9	1.000		
		**C_2**	0.3	1.000	1.3	1.000	0.3	1.000		
		**C_3**	0.6	1.000	2.8	0.643	0.6	1.000		
		**C_4**	1.4	1.000	6.4	**< 0.0001**	0.6	1.000		
		**C_5**	0.9	1.000	16.4	**< 0.0001**	0.0	1.000		
	48 h	**Cnt**	1.6	1.000	2.0	0.985	2.5	0.667		
		**C_1**	0.6	1.000	0.9	1.000	5.6	**< 0.0001**		
		**C_2**	0.4	1.000	2.4	0.902	4.8	**0.001**		
		**C_3**	1.2	1.000	6.8	**< 0.0001**	3.5	0.092		
		**C_4**	2.2	0.968	9.0	**< 0.0001**	3.5	0.086		
		**C_5**	4.9	**0.001**	15.3	**< 0.0001**	3.9	**0.025**		
CT vs DTF_H	24 h	**Cnt**	1.7	0.999	1.8	0.998			2.4	0.744
		**C_1**	6.0	**< 0.0001**	0.4	1.000			2.6	0.584
		**C_2**	2.9	0.567	1.5	1.000			2.1	0.924
		**C_3**	3.4	0.186	0.3	1.000			2.1	0.924
		**C_4**	1.6	1.000	3.5	0.146			2.1	0.891
		**C_5**	1.7	0.999	9.0	**< 0.0001**			0.4	1.000
	48 h	**Cnt**	0.6	1.000	0.7	1.000			0.3	1.000
		**C_1**	3.1	0.365	4.7	**0.003**			0.6	1.000
		**C_2**	2.5	0.857	3.3	0.252			0.9	1.000
		**C_3**	2.0	0.991	1.2	1.000			1.4	0.999
		**C_4**	1.2	1.000	0.02	1.000			0.8	1.000
		**C_5**	2.3	0.923	6.2	**< 0.0001**			0.4	1.000
DTF_L vs DTF_H	24 h	**Cnt**	1.1	1.000	1.4	1.000				
		**C_1**	2.4	0.884	0.0	1.000				
		**C_2**	2.2	0.951	2.8	0.619				
		**C_3**	3.7	0.083	2.5	0.839				
		**C_4**	3.0	0.478	2.9	0.582				
		**C_5**	2.6	0.764	7.4	**< 0.0001**				
	48 h	**Cnt**	2.2	0.960	2.8	0.649				
		**C_1**	2.6	0.792	5.6	**< 0.0001**				
		**C_2**	2.9	0.560	5.7	**< 0.0001**				
		**C_3**	3.2	0.324	5.6	**< 0.0001**				
		**C_4**	3.4	0.219	9.0	**< 0.0001**				
		**C_5**	2.5	0.822	9.2	**< 0.0001**				

Table S4. Lethal concentrations (LC_{50} and LC_{90}) of Btk on SWD at 48 h for all temperature conditions, calculated by Probit analysis with 95% confidence intervals ($CI_{95\%}$). Significant p-values are shown in bold.

	**LC_{50}**	**$CI_{95\%}$ - LC_{50}**	**LC_{90}**	**$CI_{95\%}$ - LC_{90}**	**χ^2**	**p**
**CT**	4876	4363-5445	10713	9693-12038	185	**< 0.0001**
**DTF_L**	6768	6039-7589	15520	13980-17543	176	**< 0.0001**
**DTF_H**	3915	3286-4558	11800	10548-13463	156	**< 0.0001**
